# Metabolite Profiling and Antioxidant Activity of 10 New Early- to Mid-Season Apple Cultivars and 14 Traditional Cultivars

**DOI:** 10.3390/antiox9050443

**Published:** 2020-05-20

**Authors:** Inhwan Kim, Kyung-Hyung Ku, Moon-Cheol Jeong, Soon-Il Kwon, Jihyun Lee

**Affiliations:** 1 Department of Food Science and Technology, Chung-Ang University, Anseong 17546, Korea; hgodos@hanmail.net; 2 Korea Food Research Institute, Wanju 55365, Korea; khku@kfri.re.kr (K.-H.K.); mcjeong@kfri.re.kr (M.-C.J.); 3 Apple Research Institute, National Institute of Horticultural & Herbal Science, Rural Development, Gunwi 39000, Korea; topapple@korea.kr

**Keywords:** apple peel and pulp, phenolic, UHPLC-(ESI)-qTOF, industrial application of apple, epicatechin, chlorogenic acid

## Abstract

Early- to mid-season apple cultivars have recently been developed in response to global warming; however, their metabolite compositions remain unclear. Herein, metabolites, such as free sugars, and organic acids and antioxidant activity were determined in 10 new and 14 traditional apple cultivars. Additionally, the phenolic profiles of the apple pulp and peel were characterized by high-resolution mass spectrometry. Major phenolic compounds in apples varied depending on the cultivar and tissue (i.e., peel or pulp). Among the new apple cultivars, Decobell and Tinkerbell, showed high antioxidant activity and contained higher phenolic compound content than other cultivars in the peel and pulp, respectively. Honggeum showed high phenolic content with similar sugar to acid ratio compared to popular traditional cultivars. In addition to antioxidant phenolic contents, metabolite profile information can be used to select apple cultivars for various purposes. For example, Indo can be selected for sweet apple taste because of its higher sugar to acid ratio. This information can be used to select apple cultivars for various purposes. For example, Decobell peel could be used as sources of food supplements and food additives, and Tinkerbell pulp can be utilized for apple juice making according to its metabolite profile.

## 1. Introduction

Apples are the third most widely consumed fruit globally; approximately 83 million tons were consumed in 2017 [1]. Global warming has resulted in a rapid increase in the average surface temperature of the earth, which increased by 0.6–0.9 °C from 1906 to 2005 [2]. The rate of increase has doubled in the last 50 years, accelerating the blooming of apples [3]. Because of the warmer growing seasons, early- to mid-season apple cultivars have recently been widely produced. When traditional apples are grown at high temperatures, the fruit size increases whereas firmness decreases, decreasing the quality of the fruit [4]. Small apples are preferred for consumption as a single serving by consumers, and early- to mid-season apple cultivars have smaller fruit than traditional late season cultivars [5,6]. Furthermore, the sweet taste tends to be higher in earlier blooming apples [6], and the sweetness of the apple is correlated to its free sugar levels [7]. For example, the full bloom date of Fuji apple grown in Nagano, Japan decreased from approximately 126 days to 121 days between 1970 and 2010. However, the soluble solid content of Fuji apples grown in Nagano, Japan increased from approximately 14.5 to 15.5 °brix between 1970 and 2010 [6].

Apples are rich sources of phenolic compounds known as antioxidants. Epidemiology studies showed that the consumption of foods with high content of phenolic compounds may prevent cardiovascular disease and cancer [8]. The phenolic compositions of apples differ by cultivar and tissue (i.e., peel or pulp) [9,10]. For example, the total phenolic compound level varied widely in 12 apple cultivars (1157–5119 μg/g in the peel; 423–1534 μg/g in the pulp) [9]. Quercetin-3-*O*-galactoside, quercetin-3-*O*-glucoside, catechin, phloridzin, and cyanidin-3-*O*-galactoside were dominant phenolics in apple peel [11]; whereas chlorogenic acid, epicatechin, phloridzin, and protocatechuic acid were major phenolics found in apple pulp [12,13]. In addition to these factors, which are affected by genetic differences, the chemical composition may be affected by the growing environment, postharvest techniques, and agricultural practices [14,15,16]. For example, the level of quercetin-3-*O*-galactoside in the peel of Fuji apple grown under a hail net and the orchard floor was covered with reflective foil (603 mg/kg FW) was 7.1-fold higher than that grown without the treatment (84 mg/kg FW) [17]. Additionally, we previously showed that Fuji apple peel treated with 1-methylcyclopropene (1-MCP) had significantly higher phenolic content (911 mg/kg DW) than untreated Fuji peel (796 mg/kg DW) after long-term storage (*p* < 0.05) [16]. Because various factors affect the phenolic composition of apples, it is difficult to compare the phenolic compound levels reported in different studies.

We previously examined several new early- to mid-season apple cultivars harvested in 2015 [9]. In the current study, we investigated the metabolite compositions of a larger number of apple cultivars (10 new early- to mid-season cultivars, 11 traditional early- to mid-season cultivars, and 3 traditional late-season cultivars) than the previous study (6 new early- to mid-season cultivars, 3 traditional early- to mid-season cultivars, and 3 traditional late-season cultivars). Especially, the phenolic composition of 10 apple cultivars (Summer Prince, Tinkerbell, Green Ball, Honggeum, Sansa, Sekaiichi, Alps Otome, Kamhong, Indo, Ralls Janet) were analyzed for the first time. Recently, small apples including Alps Otome apples have been preferred by consumers; however, there is little information about the chemical composition of the small apple cultivars. In our previous study, the phenolic composition of only the apple peel was investigated by ultra-high-pressure liquid chromatography coupled to accurate mass quadrupole time-of-flight MS with electrospray ionization (UHPLC-(ESI)-qTOF); thus, a limited number of phenolic compounds were identified and quantified in apple pulp by using HPLC. However, in this study, the phenolic composition of both the peel and pulp was analyzed by UHPLC-(ESI)-qTOF. Because the apple samples in this study were harvested one year after those used in our previous study in the same research orchard, yearly differences in apple metabolites were evaluated. We also expanded the scope and performing a more complete and comprehensive study, including not only more cultivars, but also a multivariate statistical analysis to differentiate the apple cultivars in terms of metabolites.

Herein, we investigated the metabolite (i.e., phenolics, free sugars, organic acids) compositions of 10 new early- to mid-season apple cultivars, 11 traditional early- to mid-season cultivars and 3 traditional late-season cultivars. The phenolic composition of both the peel and pulp was analyzed by UHPLC-(ESI)-qTOF. In addition to the metabolite compositions, antioxidant activity was determined in the apple samples. We proposed new apple cultivars for various purposes (e.g., pink pulp, high phenolic content with high sugar to acid ratio, etc.). For example, apple cultivars with high phenolic contents in the peel can be used for food supplements and food additives; apple cultivars with high phenolic contents in the pulp may be utilized for the juice making procedure.

## 2. Materials and Methods

### 2.1. Chemicals and Reagents

Authentic phenolic standards of chlorogenic acid, catechin, epicatechin, quercetin-3-*O*-galactoside, rutin, quercetin-3-*O*-glucoside, quercetin-3-*O*-xyloside, quercetin-3-*O*-arabinoside, quercetin-3-*O*-rhamnoside, isorhamnetin-3-*O*-glucoside, quercetin, kaempferol, eriodictyol-7-*O*-glucoside, cyanidin-3-*O*-galactoside, cyanidin-3-*O*-arabinoside, cyanidin-3-*O*-rhamnoside, and phloridzin were purchased from Extrasynthese (Genay, France) or Sigma-Aldrich (St. Louis, MO, USA). Sugars (glucose, fructose, sucrose, and sorbitol) and organic acids (citric acid, malic acid, and shikimic acid) were also purchased from Sigma-Aldrich. Acetonitrile (HPLC-grade) was obtained from J.T. Baker (Phillipsburg, NJ, USA). Nanopure water used in the study was obtained from a water purification system (Milli-Q Direct 8, Merck Millipore, Billerica, MA, USA).

### 2.2. Apple Samples

**Figure 1:**
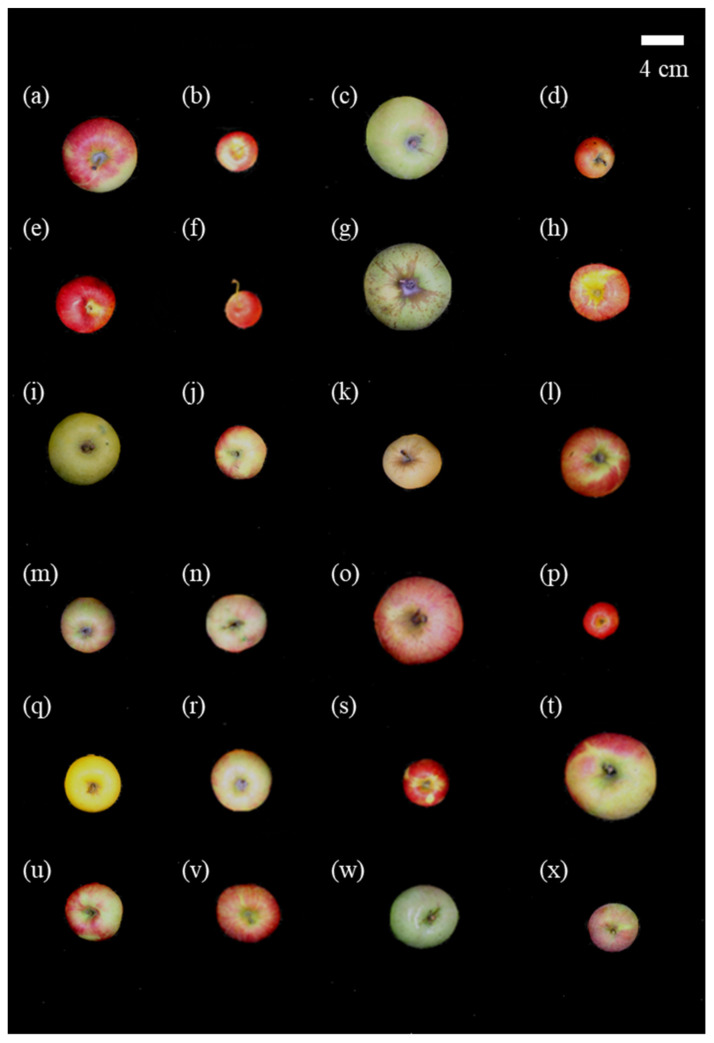
Images of apples of the 24 apple cultivars (**a**) Summer Prince, (**b**) Ruby-S, (**c**) Summer King, (**d**) Tinkerbell, (**e**) Arisoo, (**f**) Decobell, (**g**) Green Ball, (**h**) Honggeum, (**i**) Hwangok, (**j**) Picnic, (**k**) Gala, (**l**) Sansa, (**m**) Tsugaru, (**n**) Hongro, (**o**) Sekaiichi, (**p**) Alps Otome, (**q**) Golden Delicious, (**r**) Jonagold, (**s**) Jonathan, (**t**) Kamhong, (**u**) Yoko, (**v**) Fuji, (**w**) Indo, and (**x**) Ralls Janet.

Twenty-four apple cultivars were harvested from a research orchard of the Apple Research Institute, National Institute of Horticultural and Herbal Science, Gunwi (36°16′39.9″ N, 128°27′47.8″ E) Gyeongsangbuk-do, Korea, in 2016. The apple samples were listed in Table 1. In this study, a total of 24 apple cultivars were used for chemical analysis; sample information, including peel color, breeding year, genetic information, harvest season, fruit weight, and total soluble solid content, is presented in Table 1. Apple cultivars, registered since 2004, were categorized as new cultivars, whereas apples registered as far back as 1800 were categorized as traditional cultivars. Ten new early- to mid-season cultivars, 11 traditional early- to mid-season cultivars, and 3 traditional late season cultivars were evaluated. The soluble solid content ranged from 10.0 to 16.3 °Brix in the 24 cultivar apples. Jonagold apples showed the highest solid soluble content (*p* < 0.05). Images of the apple samples are shown in Figure 1.

Rootstock of Summer King, Ruby-S, Arisoo, Decobell, Hwangok, and Picnic were M.9 and 4–8 years old. The rootstock of other cultivars were M.26 and 15 years old. The apple trees were planted in on a sandy loam soil. Per unit area of 10 m^2^, 125 apple trees were planted. Apple ripeness was evaluated by the starch index and in combination with red coloration for pigmented apple cultivars. The starch index was determined by spraying a potassium-iodine solution (1.5% KI, 0.6% I) on the cut surface of apple fruits. An apple fruit was considered fully ripened when starch was completely degraded. Apple fruits were harvested when peel color of each fruit was similar to 70% levels to peel color of the fully ripened apples. Apples more than 160 fruits of each apple cultivar were collected and apple fruits without any obvious damage were collected to make a composite sample. Apple fruits were peeled to separate the peel and pulp. To make a composite sample for the pulp, after peeling, each apple fruit was sliced into six pieces and only two pieces were used for making a composite sample for analyses of phenolics and antioxidant activity; and the other two pieces were used for making another composite sample for sugar and acid analysis that require fresh fruits. The apple samples for analyses of phenolic composition and antioxidant activity were lyophilized and the dried apple samples were stored at −80 °C until analysis. A composite sample of each cultivar was used for analysis.

### 2.3. Total Soluble Solid Content

Fresh apple juice produced using a quarter of selected apple was used to analyze the total soluble solid content. Total soluble solid content was analyzed using a refractometer (PAL-1, ATAGO, Tokyo, Japan). Measurement of total soluble solid content was done in triplicate (n = 3).

### 2.4. Free Sugar and Organic Acid Content of Apple Juice

Free sugar and organic acid content was determined as previously described [16]. Briefly, apple juice from the 24 cultivars was centrifuged at 11,124× *g* for 15 min. The supernatant was filtered through a 0.22-µm polyvinylidene fluoride syringe filter. Free sugars in the apple juice were analyzed by HPLC (1260 Infinity LC system, Agilent Technologies, Santa Clara, CA, USA) coupled with a refractive index detector. Ten microliters of sample were injected and free sugars were separated on a YMC-Pack Polyamine II column (4.6 × 250 mm, 5 μm, YMC, Kyoto, Japan) maintained at 35 °C. The mobile phase of water and acetonitrile at a ratio of 25:75 (*v*/*v*) was isocratically eluted at a flow rate of 1000 µL/min.

Organic acids in the apple juice were detected by HPLC (1260 Infinity II LC System, Agilent Technologies) coupled with a photo diode array (PDA) detector (G1115A, Agilent Technologies, Santa Clara, CA, USA). The injection volume was 20 µL. A Prevail Organic Acid column (4.6 × 150 mm, 5 μm, Hichrom, Berkshire, UK) was used as the stationary phase. The column temperature was maintained at 30 °C. The mobile phase of 25 mM K_2_HPO_4_ (pH 2.8) was isocratically eluted at a flow rate of 800 µL/min. The detection wavelength was 210 nm. Measurement of free sugar and organic acid level were done in triplicate (n = 3).

### 2.5. Extraction Procedure for Analyzing Antioxidant Activities and Phenolic Composition of Apple Samples

Freeze-dried powders (0.5 g) of peel and pulp were extracted with 10,000 µL of 80% aqueous methanol solution, as described previously [9]. The mixtures were sonicated for 15 min in a sonicator (CPX3800, Branson Ultrasonics Corporation, Danbury, CT, USA), followed by centrifugation at 11,124× *g* for 15 min. The supernatant was collected to analyze antioxidant activity. To determine the phenolic composition by UHPLC-(ESI)-qTOF and HPLC coupled with a PDA detector, the supernatants were concentrated on a Speedvac concentrator (SPD 2010, Thermo Scientific, Waltham, MA, USA). The concentrates were reconstituted with 1000 µL of the initial mobile phase. The dissolved concentrates were filtered through a 0.22-µm polyvinylidene fluoride filter prior to analysis. The extraction for each analysis except for analysis of phenolic compounds composition using UHPLC-(ESI)-qTOF were done in triplicate. The extraction for phenolic composition of apple peel and pulp using UHPLC-(ESI)-qTOF was done in duplicate (n = 2).

### 2.6. Antioxidant Activities

Antioxidant activities were evaluated as the 2,2-diphenyl-1-picrylhydrazyl radical scavenging activity and 2,2′-azino-bis(3-ethylbenzothiazoline-6-sulphonic acid) radical scavenging activity. 2,2-Diphenyl-1-picrylhydrazyl radical scavenging activity was evaluated as described previously [18], with some modifications. Extracts of the peel and pulp (150 µL) were reacted with 200 µL of DPPH dissolved in ethanol. The absorbance was measured at 517 nm after 15 min.

2,2′-Azino-bis(3-ethylbenzothiazoline-6-sulphonic acid) (ABTS) radical scavenging activity was evaluated as described previously [19]. Briefly, ABTS dissolved in phosphate buffer was added to potassium persulfate solution. After incubation for 24 h, 300 µL ABTS solution was mixed with 40 μL of apple sample extracts. The absorbance at 734 nm was measured after 10 min. Antioxidant activity data were expressed as mg Trolox equivalent/g DW.

### 2.7. Determination of Phenolic Compounds Using UHPLC-(ESI)-qTOF and HPLC-PDA Detector

Phenolic compounds in the apple peel and pulp were identified as described in our previous study by UHPLC-(ESI)-qTOF (Agilent 1290 Infinity UHPLC coupled to a 6530 Series qTOF LC/MS) [9]. Blank/solvent runs were tested for background testing every ten sample runs. Phenolic compounds in apple samples were separated on a Poroshell C_18_ column (2.1 × 100 mm, 2.7 µm, Agilent Technologies, Santa Clara, CA, USA) at 30 °C. The mobile phase was 0.1% formic acid in water (A) and 0.1% formic acid in acetonitrile (B) at a flow rate of 400 µL/min. The gradient of the mobile phase was as follows: 5–10% B, 0–5 min; 10–12% B, 5–8 min; 12–15% B, 8–10 min; 15% B, 10–15 min; 15–55% B, 15–18 min; 55–90% B, 18–20 min; and 90–5% B, 20–22 min. The column temperature was maintained at 30 °C. The sample injection volume was 5 µL. For all samples, both negative and positive ionization modes were used. Mass scanning was conducted over range of *m*/*z* 100–1000. The drying gas heated to 225 °C was supplied at a rate of 8 L/min. The capillary, fragmentor, and skimmer voltage were 3.5 kV, 125 V, and 65 V, respectively. A phenolic compound database was developed that contained possible phenolic compounds from previous literature. The database contained theoretical *m*/*z* of phenolic compounds based on their molecular formula. Identification of phenolic compounds was conducted by comparing observed *m*/*z* with theoretical *m*/*z*. Mass error was calculated and used as cut-off (10ppm). Isotope abundance and spacing were compared using Masshunter Qualitative Analysis (Agilent Technologies, Santa Clara, CA, USA). For MS/MS fragment interpretation of the phenolic compounds identified in MS1 mode, MS2 data were acquired using the auto MS/MS mode with a collision energy of 20 eV. MS2 data were obtained at an MS/MS scan rate of three spectra/second. The relative quantification of each phenolic compound in the apple peel and pulp extract was conducted by qTOF in the MS1 mode using the ion peak area extracted at its retention time.

Phenolic compounds in the apple peel and pulp were quantified by HPLC (1260 Infinity II LC System, Agilent Technologies, Santa Clara, CA, USA) coupled with a photodiode array (PDA) detector (G1115A, Agilent Technologies, Santa Clara, CA, USA). The injection volume was 20 µL. Phenolics were separated on a ZORBAX eclipse XDB C_18_ column (4.6 × 250 mm, 5 µm, Agilent Technologies, Santa Clara, CA, USA) after identification based on authentic phenolic standards, as described in our previous study [9]. The column temperature was maintained at 30 °C and the flow rate of the mobile phase was 800 µL/min. The mobile phase was 0.1% formic acid in water (A) and 0.1% formic acid in acetonitrile (B). The gradient conditions of the mobile phase were as follows: 5–10% B, 0–10 min; 10–36% B, 10–45 min; 36–95% B, 45–52 min; 95% B, 52–55 min; 95–5% B, 55–58 min; and 5% B, 58–65 min. Wavelengths of 280, 320, 360, and 520 nm were monitored to detect flavan-3-ols, phenolic acids, flavonols, and anthocyanins, respectively.

### 2.8. Statistical Analysis

Statistical analysis was conducted using SPSS statistics 23 software (SPSS, Inc., Chicago, IL, USA). Significant differences in the chemical composition among the apple fruit of the 24 cultivars were confirmed by analysis of variance at *p* < 0.05. Duncan’s test was used as a post-hoc test. Principal component analysis and hierarchical cluster analysis were performed to visualize apple sample clustering using XLSTAT (XLSTAT, ver. 2017.03, Microsoft Excel Add-in software, New York, NY, USA). The data format for principal component analysis was an observation/variables table and the PCA type was Pearson’s correlation type.

## 3. Results and Discussion

### 3.1. Free Sugar and Organic Acid Content of Apple Juice

**Figure 2:**
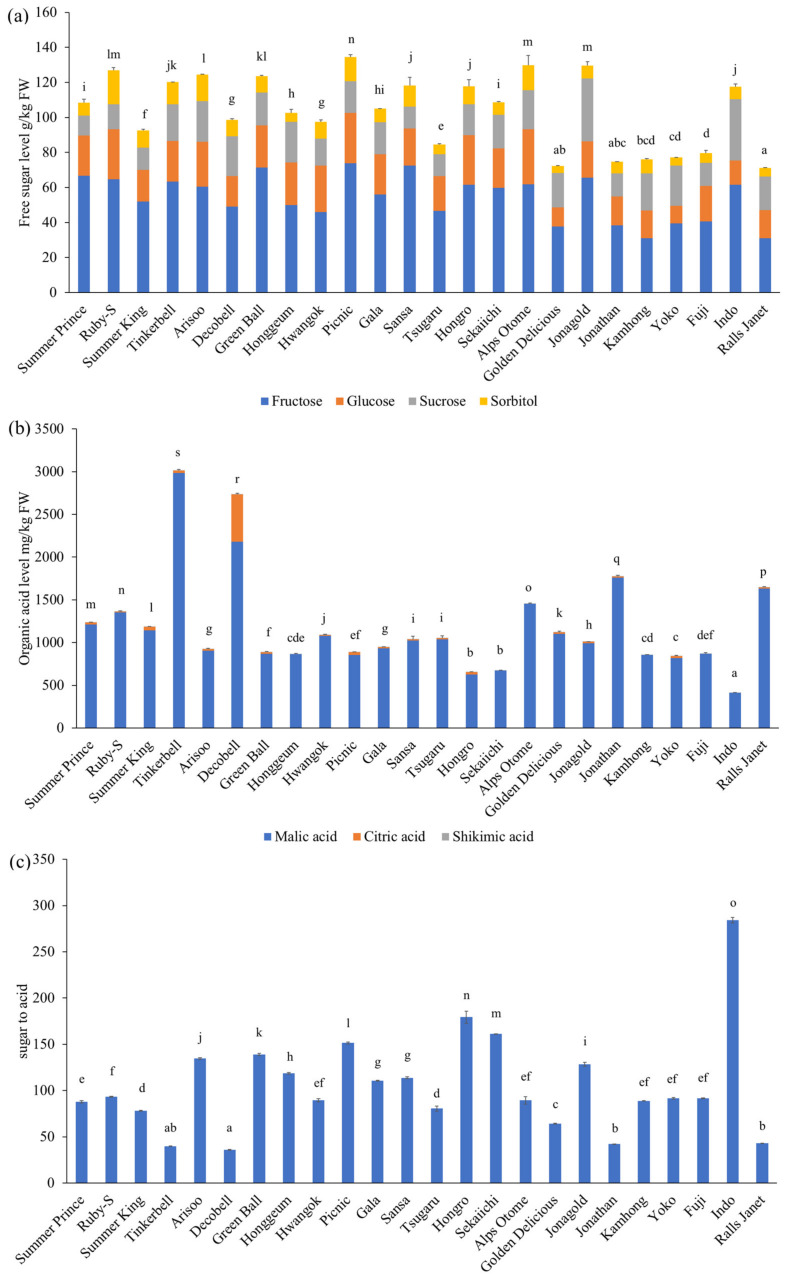
Content of free sugar (**a**), organic acid (**b**), and sugar to acid ratio (**c**) in apple juice made with 24 apple cultivars. Significant differences between cultivars at *p* < 0.05 are denoted by different letters. The standard deviation refers to the total amount of sugars and organic acids.

Free sugar and organic acid content of the 24 apple cultivars is shown in Figure 2. The total sugar content (sum of fructose, glucose, sucrose, and sorbitol) ranged from 71.2 to 134.4 g/kg fresh weight (FW), which was consistent with previously reported values of 74.7–199.2 g/kg FW [7,9]. A new apple cultivar, Picnic apples, contained a higher total sugar content (134.4 g/kg FW) than the other cultivars (*p* < 0.05). This is consistent with the results of our previous study of new apple cultivars harvested in 2015 (a year before the harvest of the apple samples in this study) [9]. The total sugar content in Fuji apples (79.7 g/kg FW) in this study was lower than that in Fuji apples grown in Europe (150 g/kg FW) [20]; moreover, it was similar to that of Fuji apples grown in New Zealand (72.2–75.3 g/kg FW) [21]. Newly developed yellow-skinned apple cultivars, Summer King and Hwangok apples, contained higher total sugar content (92.6–97.5 g/kg FW) compared to Golden Delicious apples (72.1 g/kg FW)—a commercially important apple cultivar with yellow skin (*p* < 0.05).

Fructose was the major sugar in all apple cultivars. However, the ratio of glucose and sucrose varied depending on the cultivar. For example, Fuji apples contained higher levels of glucose (20.3 g/kg FW) than sucrose (13.1 g/kg FW). However, Golden Delicious apples contained higher levels of sucrose (19.6 g/kg FW) than glucose (11.1 g/kg FW). This is important because each sugar has a different sweet taste intensity. For example, 1 g/L glucose solution is as sweet as 0.6 g/L sucrose solution. Thus, the relative sweet taste intensity of glucose solution is lower than that of sucrose solution.

Total organic acid content (sum of malic acid, citric acid, and shikimic acid) of the 24 apple cultivars ranged from 414 to 3015 mg/kg FW. A new apple cultivar, Tinkerbell, contained higher total organic acid content than the other cultivars (*p* < 0.05). Indo apples showed the lowest organic acid content (*p* < 0.05). The major organic acid in apple juice was malic acid, which ranged from 413 to 2984 mg/kg FW, followed by citric acid and shikimic acid. The organic acid composition varied according to the apple cultivar. Citric acid was not detected in Alps Otome, Honggeum, Indo, Kamhong, or Sekaiichi. However, Decobell apples had significantly higher citric acid content (551.2 mg/kg FW) than the other cultivars (*p* < 0.05). This agree with the results of our previous study [9]. We collected the same apple cultivars grown in the same area in different years. In addition to genotype, other factors, such as cropping year and agricultural practices, (e.g., organic or integrated cultivation) may affect the citric acid content [20].

The sugar to acid ratio has been used as an indicator of sweetness and sourness of apples [22]. We found approximately 8-fold variations in the sugar to acid ratio, with Indo apples showing the highest ratio. Thus, Indo apples may taste sweeter than other cultivars. Commercially important apple varieties (i.e., Fuji, Gala, and Golden Delicious apples) showed sugar to acid ratio values of 64.3–110.6 in this study. Among the new apple cultivars, Summer Prince, Ruby-S, Summer King, and Hwangok apples were in this range. Additionally, Honggeum apples showed sugar to acid ratios similar to Gala apples, one of the most popular traditional apple cultivars consumed worldwide. Honggeum apples also showed similar free sugar content and organic acid content to Gala and Fuji apples.

### 3.2. Antioxidant Activity

Antioxidant activity (DPPH and ABTS) was investigated in the peel and pulp of the 24 apple cultivars (Table 2). In general, the apple peel showed higher antioxidant activity than the apple pulp, regardless of the cultivar. This agrees with the results of our previous study [9]. Among apple cultivars, both the peel and pulp of Decobell apples showed higher antioxidant activity in the DPPH assay (1.25 and 0.87 mg te/g DW for peel and pulp, respectively) and ABTS assay (7.70 and 4.20 mg te/g DW for peel and pulp, respectively) than the other cultivars (*p* < 0.05). The antioxidant activity of the peel of Decobell apples showed 5.5% higher antioxidant activity compared to that of Decobell apples harvested in 2015 in the ABTS assay (7.30 mg te/g DW) [9]. From 2015 to 2016, the average temperature increased from 25.1 °C to 26.1 °C in July; from 25.5 °C to 27.3 °C in August; and from 20.4 °C to 21.4 °C in September [1]. Therefore, it seemed that the increased temperature increases antioxidant activity possibly due to heat stress [23].

### 3.3. Identification of Phenolic Compounds in the Apple Peel and Pulp by UHPLC-(ESI)-qTOF

Phenolic compounds in both the apple peel and pulp of all cultivars were identified by UHPLC-(ESI)-qTOF (Table S1). Positive mode was used to analyze anthocyanin, whereas negative mode was used to analyze the remaining phenolic compounds, as the positive and negative modes show better responses for anthocyanins and other phenolics, respectively [9]. Figure S1 shows the extracted ion chromatograms of phenolic compounds identified in the MS1 subset data of Decobell apple peel and pulp in positive mode for anthocyanins and in negative mode for other phenolics.

Phenolic compounds were identified based on their accurate mass by comparing their high-resolution mass spectrometry data obtained in MS1 mode of qTOF with the calculated masses and isotopic patterns. Peaks showing mass errors of less than 5.1 ppm were further confirmed by tandem mass spectrometry (qTOF MS/MS). If authentic standards were available, the identification results were confirmed.

In the apple peel, 47 phenolic compounds were characterized in the 24 apple cultivars, including 7 phenolic acids, 12 flavan-3-ols, 18 flavonols, 2 flavanones, 6 anthocyanins, and 2 dihydrochalcones. In the apple pulp, 19 phenolic compounds were identified, including 4 phenolic acids, 9 flavan-3-ols, 3 flavonols, 1 flavanone, 1 anthocyanin, and 1 dihydrochalcone. In our previous study, only five phenolic compounds (i.e., catechin, epicatechin, quercetin-3-*O*-rhamnoside, phloridzin, and chlorogenic acid) were identified in the apple pulp by HPLC [9]. In this study, fourteen phenolic compounds were additionally identified in the apple pulp by using UHPLC-(ESI)-qTOF.

The phenolic compounds were tentatively identified based on accurate mass and MS/MS spectra. The identity of 17 phenolic compounds was confirmed using authentic standards, as marked in the Table S1. The mass fragmentation patterns of each phenolic compound was described in our previous study [9]. In our previous work, the qTOF MS2 spectra of several phenolics such as hydroxybezoic acid, dihydroxy benzoic acid and procyanidin trimer were not observed—possibly due to the low abundance of the precursor ion [9]; however, that was found in this study.

Phenolic acids identified in the apple peel/pulp included two hydroxybenzoic acids and five hydroxycinnamic acids. Hydroxy benzoic acid (Table S1, peak PA1) produced fragment ions, likely produced by neutral loss of the H_2_O or CO moiety. Dihydroxy benzoic acid (Table S1, peak PA2) produced fragment ions corresponding to the loss of the CO or CO_2_ moiety. Flavan-3-ol monomers (i.e., catechin, epicatechin, (epi)gallocatechin, and catechin-*O*-hexoside isomers) and complex polymers of flavan-3-ol monomers (i.e., proanthocyanidins) were identified in the peel and/or pulp of apple samples. The fragment ion at m/z 577.1350 from the procyanidin trimer (Table S1, peak P10) was identified as the (epi)catechin-(epi)catechin dimer. Eleven quercetin glycosides were identified in the apple samples (Table S1, peaks F2–F10, F12, F16). Most flavonols and flavanones were detected in the peel of the apples rather than in the pulp. However, quercetin-3-*O*-galactoside, quercetin-3-*O*-glucoside, quercetin aglycone, and naringenin-*O*-hexoside were present in both tissues (i.e., peel and pulp) of the apple samples. All identified anthocyanins (Table S1, peaks A1–A6) were cyanidin glycosides. In this study, compared to previous studies, cyanidin-3-*O*-rhamnoside (Table S1, peak A6) was identified for the first time in the apple peel [24,25,26,27,28]. A dihydrochalcone, phloridzin was found in the peel and pulp of apples.

### 3.4. Relative Quantification of Phenolic Compounds in the Apple Peel and Pulp by UHPLC-(ESI)-qTOF

**Figure 3:**
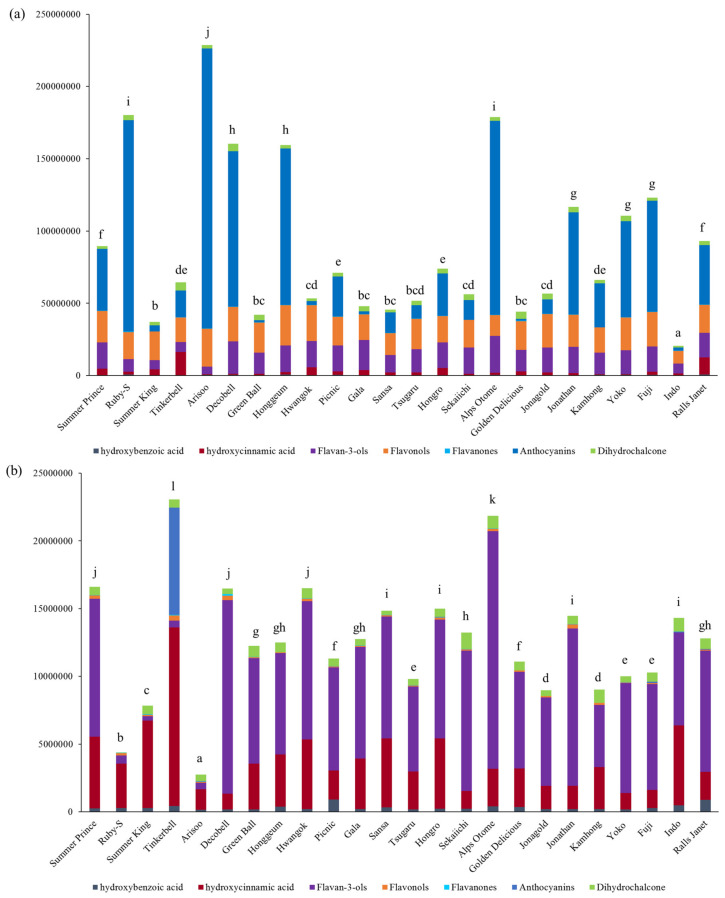
Average extracted ion chromatogram (EIC) peak areas of the identified phenolic compounds obtained from MS1 data of UHPLC-(ESI)-qTOF in (**a**) peel and (**b**) pulp of apple samples. Significant differences between cultivars at *p* < 0.05 are denoted by different letters.

Because authentic standards of most phenolic compounds identified in this study are not commercially available, the phenolic compounds in peel and pulp were relatively quantified using extracted ion chromatogram peak areas from the MS1 data of UHPLC-(ESI)-qTOF. The results are shown in Figure 3. For a simple interpretation of the MS1 data, the 47 identified phenolic compounds were grouped into the subclass: hydroxybenzoic acid, hydroxycinnamic acid, flavan-3-ols, flavonols, flavanones, anthocyanins, and dihydrochalcone as shown in Table S1.

In the apple peel, anthocyanins and flavonols were predominant phenolic compound subclasses. However, the peel of several apple cultivars (i.e., Summer King, Green Ball, Hwangok, Gala, Golden Delicious, Jonagold, and Indo) contained flavonols and flavan-3-ols as predominant subclasses of phenolic compound. Arisoo peel contained highest anthocyanin levels as compared to other cultivars investigated (*p* < 0.05). Among new apple cultivars, Ruby-S, Arisoo, Decobell, and Honggeum peel showed significantly higher sum of peak areas for the identified compounds than Golden Delicious, Gala, and Fuji peel—the most popular traditional apple cultivars consumed worldwide (*p* < 0.05).

All investigated apple cultivar pulp except for Tinkerbell were composed of flavan-3-ols and hydroxycinnamic acid as predominant subclasses of phenolic compound. Tinkerbell pulp contained flavan-3-ols and anthocyanin as predominant subclasses of phenolic compound. The high contents of anthocyanins possibly contributed to the red color flesh of Tinkerbell apples. Alps Otome pulp had highest flavan-3ols levels than other cultivar pulp. Among new apple cultivars, Summer Prince, Tinkerbell, Decobell, and Hwangok showed significantly higher sum of peak areas for the identified compounds than Golden Delicious, Gala, and Fuji pulp (*p* < 0.05). In our previous study, the phenolic composition of only the apple peel was investigated by ultra-high-pressure liquid chromatography coupled to accurate mass quadrupole time-of-flight MS with electrospray ionization. Thus, we cannot compare the pulp result; however, the phenolic composition in the peel of apple cultivars in this study agrees with that in our previous study for the same apple cultivars (i.e., Ruby-S, Summer King, Arisoo, Decobell, Hwangol, Picnic, Hongro, Yoko, Fuji) [9].

### 3.5. Quantification of Phenolic Compounds in the Apple Peel and Pulp by HPLC-PDA

Phenolic compounds in peel and pulp were quantified using an HPLC system coupled with a PDA detector; the results are shown in Table 3. Total phenolic compound contents (sum of the individual phenolic compound content) varied depending on the cultivar and tissue (i.e., peel or pulp). These values ranged from 672 to 2699 µg/g DW in apple peel and from 246 to 1741 µg/g DW in apple pulp. Decobell and Tinkerbell contained higher phenolic compound content than other cultivars in the peel and pulp, respectively. Based on the data obtained by UHPLC-(ESI)-qTOF, Arisoo and Tinkerbell showed the highest phenolic compound content than other cultivars in the peel and pulp, respectively. The disagreement between the results obtained by HPLC-PDA and UHPLC-(ESI)-qTOF may be explained by the differences in the ionization efficiency of each individual phenolic compounds in UHPLC-(ESI)-qTOF analysis.

Major phenolic compounds in apples varied depending on the tissue (i.e., peel or pulp). The results of our previous study and those of other previous studies revealed that epicatechin and quercetin conjugates are the major phenolic compounds in the peel, whereas (epi)catechin and chlorogenic acid are the predominant phenolic compounds in apple pulp [9,11,29]. The results of most apple cultivars in this study agreed with those of previous studies.

Predominant phenolic compounds in apple peel varied with the cultivar as well. Interestingly, several new apple cultivars (Ruby-S, Tinkerbell, and Picnic) contained chlorogenic acid as the major phenolic compound in the peel (approximately 45% of total). Chlorogenic acid has been reported to have the second highest alkyl peroxy radical scavenging activity after rutin among the 18 other flavonoids and phenolic compounds, which may explain the anticancer activity of apples [30]. The quercetin conjugate quercetin-3-*O*-galactoside was the major phenolic compound in 10 cultivar apple peels (Arisoo, Decobell, Fuji, Green Ball, Hwangok, Kamhong, Sansa, Summer King, Summer Prince, and Tsugaru), ranging from 51 (Alps Otome) to 726 (Arisoo) µg/g DW; this result agrees with that of a previous study showing a range of 80–1909 µg/g DW in 15 apple cultivars including Braeburn, Elstar, Golden Delicious, Granny Smith and Jonagold grown in Germany [31]. Gala, Golden Delicious, and Fuji, the most popular traditional apple cultivars consumed worldwide, contained 136–344 µg/g DW of quercetin-3-*O*-galactoside levels. Among the new cultivars, Summer Prince, Arisoo, Decobell, Honggeum, and Hwangok (422–726 µg/g DW) contained significantly higher quercetin-3-*O*-galactoside levels in the peel than Gala, Golden Delicious, and Fuji apples (*p* < 0.05). In the peel, Golden Delicious apples in this study contained a higher quercetin-3-*O*-galactoside level (188 µg/g DW) than Golden Delicious apples grown in the USA (42 µg/g DW) [32] and a lower quercetin-3-*O*-galactoside level than Golden Delicious apple grown in Canada and China (431–725 µg/g DW), after correcting the fresh weight (FW) to the DW based on a 90% estimated moisture content [11,33].

Epicatechin and quercetin-3-*O*-rhamnoside were the major compounds in the peel of other apple cultivars. Epicatechin content ranged from 81 (Summer King) to 578 (Alps Otome) µg/g DW in the peel, corresponding to the results of a previous study that reported catechin levels of 90–620 µg/g DW in apple grown in Slovenia, after correcting the FW values to DW [13]. Gala, Golden Delicious, and Fuji apples contained 178–367 µg/g DW of epicatechin levels. Among the new cultivars, Decobell and Honggeum apples (510 and 424 µg/g DW, respectively) contained significantly higher epicatechin levels in the peel than Gala, Golden Delicious, and Fuji peels (*p* < 0.05). In the peel, Golden Delicious apples in this study contained a higher epicatechin level (188 µg/g DW) than Golden Delicious apples grown in the USA and China (24–71 µg/g DW) [32,33] and a lower epicatechin level than Golden Delicious apple grown in Canada (658 µg/g DW), after correcting the FW values to DW [11].

Major phenolic compounds in apple pulp also varied depending on the cultivar. For example, Tinkerbell apples showed high levels of anthocyanin (e.g., cyanidin-3-*O*-galactoside). The high anthocyanin contents may be responsible for the pink color of the Tinkerbell apple flesh. Decobell cultivar contained epicatechin as the major compound (approximately 46.6% of total phenolic compound content) in the pulp.

The major compound in the apple pulp was chlorogenic acid (approximately 48–93% of total phenolic compound contents) in most apple cultivars. The chlorogenic acid content ranged from 125 (Tsugaru) to 1623 (Tinkerbell) µg/g DW, which agreed with previously reported values (252–2345 µg/g DW) of apple grown in Canada, after correcting the FW values to DW [34]. Gala, Golden Delicious, and Fuji apples contained 349–523 µg/g DW of chlorogenic acid levels. Among the new cultivars, Ruby-S, Tinkerbell, Honggeum, and Picnic apples (562–1623 µg/g DW) contained significantly higher chlorogenic acid levels in the pulp than Gala, Golden Delicious, and Fuji apples (*p* < 0.05). Our Golden Delicious apple pulp contained a chlorogenic acid level (349 µg/g DW) similar to that of Golden Delicious apples grown in Spain (290–570 µg/g DW) [35]; furthermore, in the pulp, they contained a lower chlorogenic acid level than those grown in Canada (1563 µg/g DW) [11]. Our Fuji apple pulp contained a chlorogenic acid level (375 µg/g DW) similar to those grown in Korea (7–6970 µg/g DW) [36], and our Fuji apple pulp showed a lower chlorogenic acid level (375 µg/g DW) than those grown in Poland (724 µg/g DW) [37].

Epicatechin levels in the apple pulp ranged from 4 (Summer King and Tinkerbell) to 298 (Decobell) µg/g DW. This agreed with previously reported values of epicatechin in apple pulp grown in Spain (62–307 µg/g DW) [29]. Gala, Golden Delicious, and Fuji apples contained 85–99 µg/g DW of epicatechin levels. Among the new cultivars, Summer King and Decobell apples (121–298 µg/g DW) showed significantly higher epicatechin levels in the pulp than Gala, Golden Delicious, and Fuji apples (*p* < 0.05). Our Golden Delicious apple pulp contained a lower epicatechin level (85 µg/g DW) than Golden Delicious apples grown in Europe and Canada (190–658 µg/g DW) [11,35,38]. In the pulp, Fuji apples in this study contained a lower epicatechin level (99 µg/g DW) than Fuji apples grown in Poland and Italy (237–611 µg/g DW) [37,38]. Variations in the reported level of each phenolic compound for the same apple cultivar in different studies may be the result of the different maturity levels of the harvested apples, UV radiation exposure, growing temperatures, and agricultural practices [39,40,41].

Previously, we determined the phenolic compositions of apple cultivars, including six new apple cultivars (i.e., Summer King, Ruby-S, Arisoo, Decobell, Hwangok, Picnic apples), harvested in 2015 [9]. The phenolic levels in the apple peel in 2015 were higher than those in 2016. For example, the peel of Summer King apples harvested in 2015 and 2016 contained 431 and 364 µg quercetin-3-*O*-galactoside/g DW, respectively. Additionally, the epicatechin content in Hongro harvested in 2015 (1015 µg epicatechin/g DW) was 2.22-fold higher than that harvested in 2016 (457 µg epicatechin/g DW). The pulp of Decobell apples harvested in 2015 (433 µg chlorogenic acid/g DW) contained a 1.62-fold higher chlorogenic acid level than that of Decobell apples harvested in 2016 (268 µg chlorogenic acid/g DW) [9]. The average temperature of Gunwi increased from 14.4 °C to 14.6 °C from 2015 to 2016 [1]. No studies have yet reported the effect of elevated temperature on the phenolic composition of apples. However, a previous study showed that as the temperature increased, leafy phenolics, mainly flavonols and phenolic acids, of *Populus tremula* L. (European aspen) were decreased. Furthermore, organic acid levels and soluble solid content, which mainly contribute to the taste of apples, showed decreasing trends as the air temperature increased [6]. These patterns were also observed in our study.

### 3.6. Principal Component Analysis (PCA) Heatmap Result

**Figure 4:**
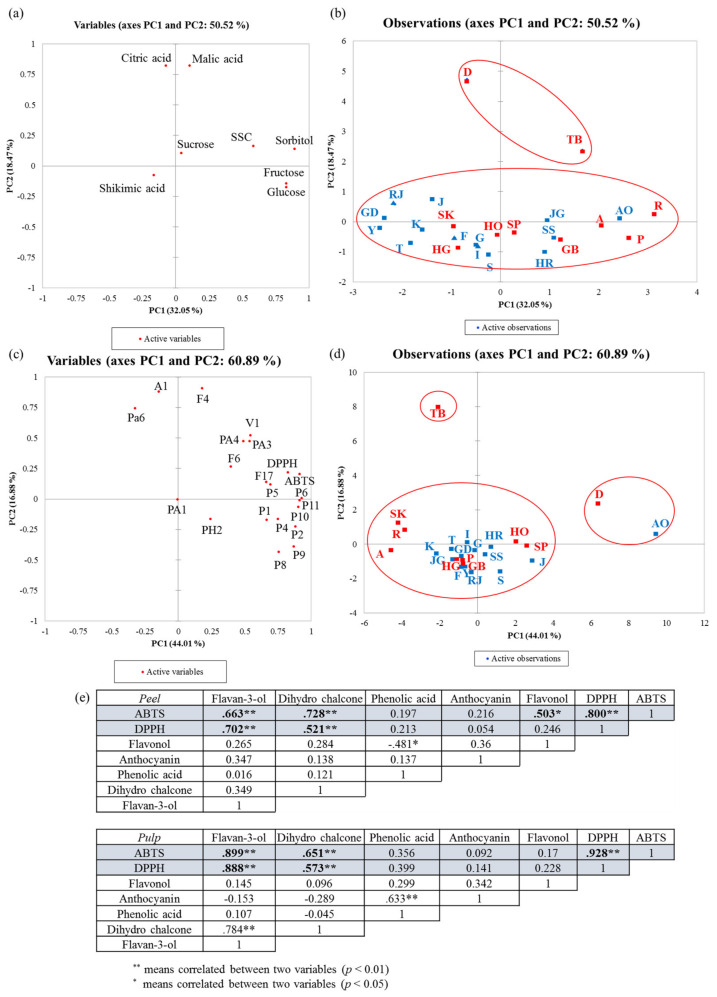
Principal component analysis of apple pulp: (**a**) PCA loading plot based on brix, organic acids, and free sugars, (**b**) PCA score plot based on brix, organic acids, and free sugars, (**c**) PCA loading plot based on phenolic compounds identified by UHPLC-(ESI)-qTOF, (**d**) PCA score plot based on phenolic compounds, (**e**) Pearson correlation matrix of results obtained from the apple samples (red and blue = new apple cultivars and traditional apple cultivars, respectively; rectangle and triangle = early- to mid-season). Sample codes for apple cultivars and compound codes correspond to those listed in Table 1 and Table S1, respectively.

Principal component analysis (PCA) was performed on the apple pulp metabolite data of 24 apple cultivars. The results of PCA based on metabolites possibly affecting the taste of apples (e.g., free sugars and acids) are shown in Figure 4a,b. Another PCA, based on metabolites possibly affecting the health benefits of apples (i.e., phenolic compounds identified by UHPLC-qTOF), is shown in Figure 4c,d.

In Figure 4a,b, the first two principal components (PCs) accounted for 50.52% of the variations. PC1 and PC2 contributed to 32.05% and 18.47% of the total variance, respectively. Decobell and Tinkerbell apples were separated from other apple samples, and the samples were spread on the upper side of PC2. These new apple cultivars were correlated with citric acid and malic acid.

In Figure 4c,d, the first two PCs accounted for 60.89% of the variations. PC1 and PC2 contributed to 44.01% and 16.88% of the total variance, respectively. Apple samples were well-separated into three clusters (cluster 1, Decobell (D) and Alps Otome (AO); cluster 2, Tinkerbell; cluster 3, for other cultivars). Cluster 1 samples (D and AO) were spread on the positive side of PC1. Cluster 1 samples were correlated with most phenolic compounds and antioxidant activity. Cluster 2 was spread on the positive side of PC2 and correlated with cyanidin-3-*O*-galactoside (A1), a coumaroylquinic acid isomer (Pa6), and quercetin-3-*O*-galactoside (F4).

Figure 4e shows the Pearson correlation results between antioxidant activities and phenolic compound levels in apple peel and pulp. To simplify the data presentation, the identified phenolic compounds were grouped into five categories (i.e., flavan-3-ol, dihydrochalcone, phenolic acid, anthocyanin, and flavonol) by summing the levels of phenolic compounds under the same category.

In apple peel, the flavan-3-ol and dihydrochalcone content was positively correlated with antioxidant activity (DPPH and ABTS) (*p* < 0.01). Flavonol content was positively correlated with only ABTS radical scavenging activity (*p* < 0.05). However, phenolic acid and anthocyanin content were not significantly correlated with antioxidant activity (DPPH and ABTS) (*p* > 0.05). Similar to that in the peel, flavan-3-ol and dihydro chalcone content was positively correlated with antioxidant activity (DPPH and ABTS) in the pulp (*p* < 0.01). Anthocyanin and flavonol levels were not significantly correlated with antioxidant activities in the pulp (*p* > 0.05). This may explain the discrepancy of the antioxidant activity data and phenolic content data. For example, Tinkerbell peel showed the highest phenolic content; however, Decobell peel had the highest antioxidant activity (*p* < 0.05). The high antioxidant activity of Decobell peel could be explained by high content of flavan-3-ols.

**Figure 5:**
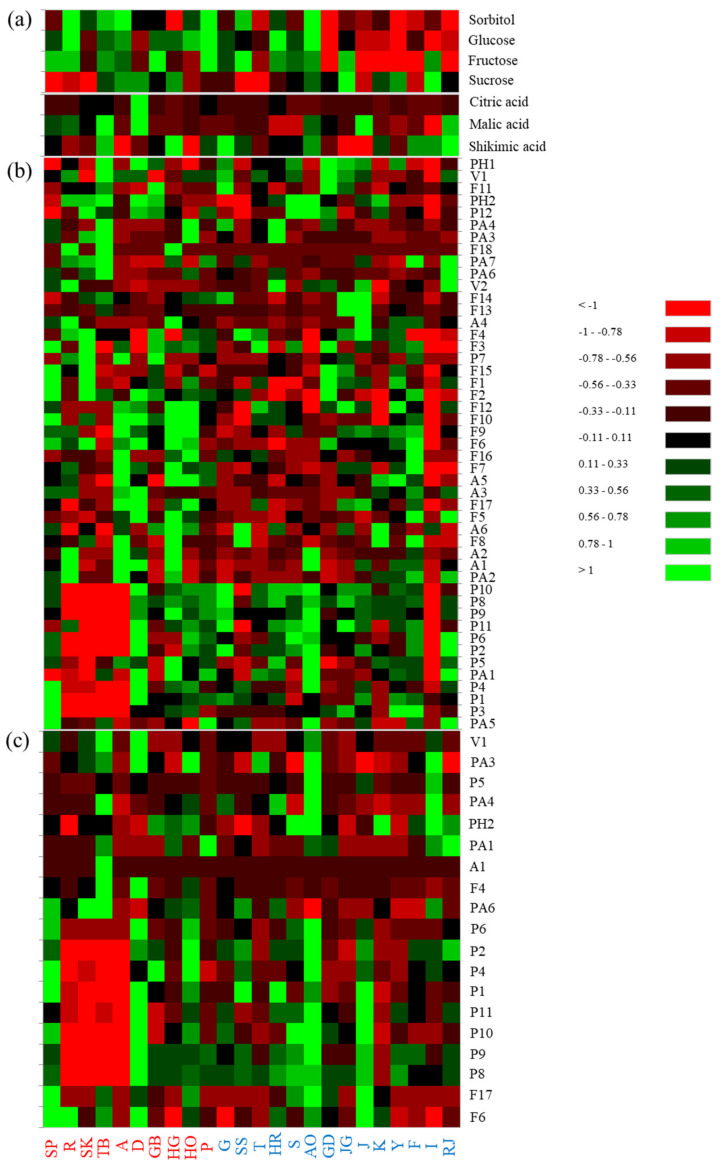
Heatmap visualization of differences in the identified apple metabolites (free sugars and organic acids (**a**), phenolics in the peel (**b**), and in the pulp (**c**)) in different apple cultivars (red and blue color in bottom of heatmap = new apple cultivars and traditional apple cultivars, respectively). Green and red colors presented in the heatmap indicate values above and below the mean centered and scaled expression values, respectively. Black indicates values close to the mean.

Figure 5a–c presents a heatmap of identified metabolites (i.e., free sugars, organic acids, phenolics) in apple samples. The data reveal that Summer Prince, Ruby-S and Picnic apples contained lower sucrose contents but higher fructose and glucose levels than other apple cultivars. Ruby-S, Summer King, Tinkerbell, Arisoo, Hwangok, and Picnic apples had higher sorbitol levels than other apple cultivars. Thus, because of the high sorbitol content, eating these apple cultivars will give a cool feeling. Decobell apples contained higher malic acid and citric acid levels than other cultivars. Tinkerbell, Hongguem and Picnic apples contained higher shikimic acid levels than other new cultivars. In the peel, Decobell apples had higher phenolic levels than other cultivars. Among new apple cultivars, Honggeum peel showed higher levels of flavan-3-ols and most quercetin conjugates than other new apple cultivars. Tinkerbell apples contained higher levels of most phenolics than other apple cultivars in the pulp. Decobell apples contained higher flavan-3-ols levels than other new cultivars in the pulp.

## 4. Conclusions

Metabolite (free sugars, free organic acids, and phenolic compounds) analysis and antioxidant activities were determined in 24 apple cultivars grown in 2016. In this study, the metabolite profiles of a larger number of apple cultivars than our previous study were performed. Especially, the phenolic composition of ten apple cultivars were investigated for the first time. Multivariate analysis was also performed in this study. An accurate MS, UHPLC-qTOF was performed to identify 47 and 19 phenolic compounds in the apple peel and pulp, respectively. We identified 14 more phenolic compounds in the apple pulp by using UHPLC-(ESI)-qTOF, compared to our previous study using HPLC only [9]. Additionally, cyanidin-3-*O*-rhamnoside was identified in the apple peel for the first time. Major phenolic compounds in apples varied depending on the cultivar and tissue (i.e., peel or pulp). Apple peel contained epicatechin and quercetin conjugates as the major phenolic compounds. Apple pulp of all cultivars contained chlorogenic acid as the major compound. Several apple cultivars showed a different phenolic profile. For example, Tinkerbell apples contained high anthocyanin contents in the pulp. This may explain the red flesh of Tinkerbell apples. Among the new apple cultivars, Honggeum apples showed high phenolic content in the pulp with similar quality characteristics in terms of sugar to acid ratios compared to the most popular apple cultivars, such as Gala, Golden Delicious, and Fuji apples. This indicates that Honggeum may have the best quality characteristics among the newly developed apple cultivars, based on sugar to acid ratio and health-promoting activities due to their high phenolic compound content. Because the new apple cultivars of Decobell and Tinkerbell apples contained significantly higher phenolic content than other apple cultivars in the peel and pulp, respectively (*p* < 0.05), they may be useful as sources of functional ingredients. Decobell apples also showed the highest antioxidant activity (*p* < 0.05). This information can be used to select apple cultivars for various purposes (e.g., red color flesh cultivar, a cultivar with highest epicatechin, a cultivar with high antioxidant activity, etc.). For example, Decobell peel could be used as sources of food supplements and food additives, and Tinkerbell pulp can be used for apple juice making according to its metabolite profile.

## Figures and Tables

**Table 1:** Basic information of the 24 apple cultivars. Harvest season of early to mid and late cultivars were categorized according to Rural Development Administration (RDA).

Cultivar	Sample Code	Peel Color	Registration Year	Genetic Information	Harvest Season	New/Traditional	Fruit Weight (g)	Soluble Solid Content (SSC, °Brix)
**Summer Prince**	SP	red	2014	Tsugaru (IT 253737) × OBIR2T47 (IT 250090)	early to mid	new	293	13.4 ± 0.0 ^f^
					(early Jul)			
**Ruby-S**	R	Red	2014	Alps Otome (IT 249906) × Sansa (IT 225509)	early to mid	new	97	15.7 ± 0.0 ^n^
					(late Aug)			
**Summer King**	SK	Yellow	2010	Fuji × Golden Delicious	early to mid	new	313	10.9 ± 0.0 ^b^
					(late Aug)			
**Tinkerbell**	TB	Red	2012	Hongro (IT225596)	early to mid	new	120	15.9 ± 0.0 ^o^
					(late Aug)			
**Arisoo**	A	Red	2011	Yoko × Shensu	early to mid	new	305	15.3 ± 0.1 ^m^
					(early Sept)			
**Decobell**	D	red	2013	Sansa (IT225509) × JayDarling (IT225830)	early to mid	new	21	13.5 ± 0.1 ^g^
					(early Sept)			
**Green Ball**	GB	green and yellow	2008	Golden Delicious × Fuji	early to mid	new	318	15.1 ± 0.1 ^l^
					(early Sept)			
**Honggeum**	HG	red	2004	Shensu × Hongro	early to mid	new	360	12.8 ± 0.0 ^d^
					(early Sept)			
**Hwangok**	HO	yellow	2009	Hongwol × Yataka Fuji	early to mid	new	242	11.9 ± 0.0 ^c^
					(late Sept)			
**Picnic**	P	red	2008	Fuji × Sansa (IT225509)	early to mid	new	218	16.1 ± 0.1 ^p^
					(late Sept)			
Gala	G	red and yellow	1960	Kidd’s Orange Red × Golden Delicious	early to mid	traditional	266	12.8 ± 0.1 ^d^
					(late Aug)			
Sansa	SS	red	1986	Gala × Akane	early to mid	traditional	239	13.7 ± 0.0 ^h^
					(late Aug)			
Tsugaru	T	red and green	1975	Golden Delicious × Jonathan	early to mid	traditional	261	10.0 ± 0.1 ^a^
					(late Aug)			
Hongro	HR	red	1988	Supr Earliblaze × Supr Golden Delicious	early to mid	traditional	282	13.2 ± 0.0 ^e^
					(early Sept)			
Sekaiichi	S	red	1974	Golden Delicious × Red Delicious	early to mid	traditional	430	12.8 ± 0.0 ^d^
					(late Sept)			
Alps Otome	AO	red	1968	−	early to mid	traditional	38	15.6 ± 0.0 ^n^
					(early Oct)			
Golden Delicious	GD	yellow	1914	−	early to mid	traditional	254	13.9 ± 0.1 ^i^
					(early Oct)			
Jonagold	JG	red and green	1968	Jonathan × Golden Delicious	early to mid	traditional	261	16.3 ± 0.0 ^q^
					(early Oct)			
Jonathan	J	red	1826	Espopus Spitzenberg seedling (found in USA)	early to mid	traditional	185	13.5 ± 0.1 ^g^
					(early Oct)			
Kamhong	K	red	1992	Supr Earliblaze × Supr Golden Delicious	early to mid	traditional	356	14.3 ± 0.0 ^k^
					(early Oct)			
Yoko	Y	red	1981	Golden Delicious × unknown pollen parent	early to mid	traditional	243	13.2 ± 0.1 ^e^
				(possibly Jonathan)	(early Oct)			
Fuji	F	red	1962	Rall’s Janet × Red Delicious	late (late Oct)	traditional	242	15.4 ± 0.0 ^m^
Indo	I	green	1868	−	late (early Nov)	traditional	247	15.2 ± 0.1 ^l^
Ralls Janet	RJ	red	1800	−	late (early Nov)	traditional	182	14.1 ± 0.0 ^j^

Bold letters indicate new early- to mid-season apple cultivars. Mean values followed by different letters indicate significant cultivar differences at *p* < 0.05.

**Table 2:** Antioxidant activities (mg Trolox equivalent/g DW) of apple peel and pulp.

	*Peel*		*Pulp*	
Variety	DPPH	ABTS	DPPH	ABTS
**Summer Prince**	0.71 ± 0.01 ^fghi^	3.87 ± 0.01 ^f^	0.48 ± 0.02 ^J^	1.95 ± 0.05 ^IJ^
**Ruby-S**	0.41 ± 0.04 ^b^	2.50 ± 0.05 ^a^	0.18 ± 0.01 ^C^	0.54 ± 0.07 ^B^
**Summer King**	0.28 ± 0.04 ^a^	2.49 ± 0.04 ^a^	0.11 ± 0.01 ^B^	0.41 ± 0.05 ^B^
**Tinkerbell**	0.92 ± 0.03 ^k^	4.98 ± 0.07 ^i^	0.56 ± 0.03 ^LM^	2.10 ± 0.05 ^K^
**Arisoo**	0.54 ± 0.03 ^d^	3.51 ± 0.13 ^de^	0.03 ± 0.00 ^A^	0.23 ± 0.06 ^A^
**Decobell**	1.25 ± 0.03 ^m^	7.70 ± 0.07 ^j^	0.87 ± 0.04 ^O^	4.20 ± 0.07 ^N^
**Green Ball**	0.56 ± 0.01 ^d^	3.26 ± 0.06 ^bc^	0.28 ± 0.04 ^D^	1.17 ± 0.04 ^D^
**Honggeum**	0.77 ± 0.00 ^j^	4.30 ± 0.03 ^g^	0.35 ± 0.02 ^EF^	1.41 ± 0.07 ^E^
**Hwangok**	0.76 ± 0.02 ^ij^	4.32 ± 0.10 ^g^	0.45 ± 0.02 ^IJ^	1.81 ± 0.03 ^FGH^
**Picnic**	0.71 ± 0.02 ^fghi^	3.56 ± 0.18 ^e^	0.53 ± 0.03 ^KL^	1.83 ± 0.01 ^FGHI^
Gala	0.73 ± 0.04 ^ghij^	4.24 ± 0.14 ^g^	0.42 ± 0.03 ^GHI^	1.75 ± 0.07 ^FG^
Sansa	0.98 ± 0.03 ^l^	4.53 ± 0.13 ^h^	0.56 ± 0.02 ^LM^	2.15 ± 0.05 ^KL^
Tsugaru	0.79 ± 0.03 ^j^	3.26 ± 0.11 ^bc^	0.38 ± 0.04 ^EFG^	1.09 ± 0.18 ^CD^
Hongro	1.23 ± 0.04 ^m^	3.93 ± 0.18 ^f^	0.63 ± 0.04 ^N^	1.85 ± 0.01 ^GHI^
Sekaiichi	0.92 ± 0.03 ^k^	4.57 ± 0.08 ^h^	0.59 ± 0.04 ^MN^	2.03 ± 0.07 ^JK^
Alps Otome	0.92 ± 0.03 ^k^	5.04 ± 0.08 ^i^	0.83 ± 0.06 ^O^	3.96 ± 0.03 ^M^
Golden Delicious	0.51 ± 0.02 ^cd^	3.34 ± 0.04 ^cd^	0.32 ± 0.02 ^DE^	1.48 ± 0.10 ^E^
Jonagold	0.65 ± 0.05 ^e^	3.98 ± 0.05 ^f^	0.44 ± 0.04 ^HIJ^	1.76 ± 0.05 ^FG^
Jonathan	0.70 ± 0.04 ^efg^	4.29 ± 0.20 ^g^	0.39 ± 0.0.4 ^FGH^	2.25 ± 0.09 ^L^
Kamhong	0.75 ± 0.02 ^hij^	3.92 ± 0.09 ^f^	0.35 ± 0.05 ^EF^	1.02 ± 0.06 ^C^
Yoko	0.66 ± 0.04 ^ef^	3.51 ± 0.21 ^de^	0.49 ± 0.02 ^JK^	1.93 ± 0.10 ^HIJ^
Fuji	0.70 ± 0.03 ^efgh^	3.82 ± 0.16 ^f^	0.37 ± 0.02 ^EF^	1.35 ± 0.13 ^E^
Indo	0.56 ± 0.02 ^d^	3.08 ± 0.15 ^b^	0.36 ± 0.01 ^EF^	1.01 ± 0.12 ^C^
Ralls Janet	0.48 ± 0.03 ^c^	3.17 ± 0.08 ^bc^	0.34 ± 0.02 ^EF^	1.70 ± 0.08 ^F^

Bold letters indicate new early- to mid-season apple cultivars. Mean values followed by different lower case and capital letters indicate significant cultivar differences for peel or pulp, respectively, at *p* < 0.05.

**Table 3:** Content of phenolic compounds in the peel and pulp of 24 apple cultivars by HPLC (µg/g DW).

	Flavan-3-ols		Dihydrochalcones	Phenolic Acids	Anthocyanins		Flavonols				
	Cat	Epi	Phl	CA	C3gal	C3ara	Rut	Q3gal	Q3glc	Q3rha	Total
***Peel***											
**Summer Prince**	36 ± 1 ^l^	213 ± 7 ^cd^	57 ± 5 ^ab^	121 ± 1 ^f^	58 ± 3 ^e^	5 ± 0 ^bcd^	10 ± 0 ^cdef^	422 ± 25 ^j^	101 ± 5 ^jkl^	151 ± 9 ^fg^	1174 ± 54 ^ef^
**Ruby-S**	20 ± 1 ^h^	166 ± 5 ^bc^	90 ± 2 ^fghi^	301 ± 12 ^k^	223 ± 5 ^j^	37 ± 1 ^j^	3 ± 0 ^ab^	190 ± 6 ^de^	66 ± 2 ^cdefg^	73 ± 2 ^a^	1171 ± 25 ^ef^
**Summer King**	34 ± 1 ^l^	81 ± 1 ^a^	88 ± 2 ^efgh^	77 ± 1 ^d^	6 ± 1 ^a^	n.d.	n.d.	364 ± 9 ^hi^	103 ± 3 ^kl^	81 ± 1 ^a^	834 ± 15 ^b^
**Tinkerbell**	7 ± 1 ^ab^	72 ± 3 ^a^	217 ± 8 ^m^	1130 ± 12 ^o^	40 ± 2 ^cd^	n.d.	12 ± 0 ^def^	134 ± 5 ^bc^	52 ± 2 ^abc^	93 ± 5 ^ab^	1757 ± 14 ^j^
**Arisoo**	7 ± 0 ^ab^	200 ± 19 ^bc^	81 ± 2 ^defgh^	50 ± 1 ^b^	337 ± 35 ^l^	51 ± 6 ^h^	35 ± 5 ^i^	726 ± 70 ^m^	133 ± 10 ^m^	256 ± 20 ^k^	1876 ± 165 ^j^
**Decobell**	66 ± 6 ^n^	510 ± 101 ^k^	338 ± 34 ^o^	94 ± 8 ^e^	195 ± 25 ^i^	14 ± 2 ^h^	158 ± 13 ^m^	584 ± 50 ^l^	512 ± 43 ^n^	229 ± 21 ^ij^	2699 ± 302 ^l^
**Green Ball**	12 ± 0 ^ef^	161 ± 2 ^bc^	146 ± 8 ^k^	48 ± 1 ^b^	n.d.	n.d.	4 ± 0 ^abc^	243 ± 21 ^fg^	85 ± 6 ^hij^	156 ± 21 ^g^	855 ± 59 ^bc^
**Honggeum**	13 ± 0 ^efg^	424 ± 6 ^j^	91 ± 2 ^ghi^	149 ± 2 ^g^	176 ± 3 ^h^	27 ± 0 ^i^	132 ± 4 ^k^	428 ± 11 ^j^	107 ± 1 ^kl^	485 ± 7 ^m^	2031 ± 21 ^k^
**Hwangok**	13 ± 1 ^fg^	266 ± 4 ^ef^	76 ± 5 ^cdefg^	30 ± 1 ^a^	n.d.	n.d.	31 ± 2 ^hi^	506 ± 9 ^k^	114 ± 4 ^l^	345 ± 3 ^l^	1381 ± 24 ^ghi^
**Picnic**	9 ± 0 ^bcd^	277 ± 7 ^efg^	74 ± 4 ^cde^	637 ± 13 ^m^	53 ± 0 ^de^	7 ± 0 ^def^	15 ± 0 ^f^	149 ± 1 ^c^	60 ± 0 ^abcde^	121 ± 4 ^cde^	1400 ± 14 ^ghi^
Gala	23 ± 1 ^ij^	367 ± 17 ^i^	47 ± 7 ^a^	254 ± 6 ^j^	2 ± 0 ^a^	n.d.	7 ± 0 ^abcde^	136 ± 5 ^bc^	49 ± 2 ^abc^	113 ± 6 ^cd^	998 ± 31 ^cd^
Sansa	36 ± 1 ^l^	262 ± 13 ^e^	67 ± 1 ^bcd^	300 ± 10 ^k^	41 ± 2 ^cd^	3 ± 0 ^ab^	15 ± 1 ^f^	497 ± 29 ^k^	111 ± 3 ^l^	107 ± 2 ^bc^	1437 ± 58 ^hi^
Tsugaru	7 ± 0 ^ab^	252 ± 2 ^de^	79 ± 0 ^cdefg^	50 ± 1 ^b^	14 ± 0 ^ab^	n.d.	6 ± 0 ^abcd^	257 ± 9 ^fg^	63 ± 1 ^bcdef^	204 ± 2 ^h^	933 ± 14 ^bc^
Hongro	27 ± 1 ^k^	457 ± 66 ^j^	107 ± 3 ^j^	261 ± 10 ^j^	37 ± 5 ^cd^	7 ± 1 ^def^	3 ± 1 ^a^	98 ± 12 ^b^	44 ± 3 ^a^	210 ± 26 ^hi^	1250 ± 107 ^efg^
Sekaiichi	25 ± 1 ^j^	369 ± 12 ^i^	254 ± 10 ^n^	93 ± 3 ^e^	25 ± 3 ^bc^	3 ± 0 ^abc^	21 ± 2 ^g^	328 ± 19 ^h^	63 ± 3 ^bcdef^	124 ± 2 ^cde^	1304 ± 31 ^fghi^
Alps Otome	44 ± 0 ^m^	578 ± 35 ^l^	141 ± 5 ^k^	848 ± 12 ^n^	282 ± 9 ^k^	26 ± 1 ^i^	9 ± 1 ^bcdef^	51 ± 1 ^a^	79 ± 3 ^fghi^	83 ± 2 ^a^	2137 ± 67 ^k^
Golden Delicious	6 ± 0 ^a^	178 ± 1 ^bc^	104 ± 2 ^ij^	210 ± 1 ^i^	n.d.	n.d.	10 ± 1 ^cdef^	188 ± 5 ^d^	84 ± 3 ^ghij^	135 ± 7 ^ef^	915 ± 17 ^bc^
Jonagold	7 ± 0 ^a^	327 ± 2 ^ghi^	97 ± 4 ^hij^	168 ± 1 ^h^	15 ± 1 ^ab^	n.d.	13 ± 0 ^ef^	228 ± 7 ^ef^	77 ± 2 ^efghi^	355 ± 13 ^l^	1287 ± 25 ^fgh^
Jonathan	11 ± 0 ^def^	330 ± 21 ^hi^	191 ± 5 ^l^	142 ± 1 ^g^	113 ± 1 ^g^	8 ± 0 ^f^	5 ± 0 ^abc^	271 ± 10 ^g^	70 ± 2 ^defgh^	248 ± 7 ^jk^	1389 ± 35 ^ghi^
Kamhong	21 ± 1 ^hi^	315 ± 14 ^fgh^	139 ± 9 ^k^	63 ± 3 ^c^	84 ± 5 ^f^	5 ± 0 ^cde^	27 ± 1 ^h^	376 ± 19 ^i^	114 ± 5 ^l^	124 ± 6 ^cde^	1266 ± 62 ^efg^
Yoko	13 ± 0 ^fg^	290 ± 6 ^efgh^	74 ± 2 ^cdef^	46 ± 1 ^b^	100 ± 5 ^g^	7 ± 0 ^ef^	21 ± 2 ^g^	260 ± 18 ^fg^	83 ± 5 ^ghi^	225 ± 8 ^i^	1120 ± 45 ^de^
Fuji	8 ± 0 ^abc^	294 ± 10 ^efgh^	64 ± 3 ^bc^	264 ± 3 ^j^	116 ± 4 ^g^	18 ± 1 ^h^	147 ± 5 ^l^	344 ± 8 ^hi^	91 ± 2 ^ijk^	114 ± 4 ^cde^	1461 ± 29 ^i^
Indo	15 ± 1 ^g^	157 ± 24 ^b^	78 ± 5 ^cdefg^	36 ± 4 ^a^	n.d.	n.d.	14 ± 3 ^f^	158 ± 21 ^cd^	53 ± 6 ^abcd^	159 ± 20 ^g^	672 ± 87 ^a^
Ralls Janet	10 ± 0 ^cde^	207 ± 4 ^bcd^	63 ± 2 ^bc^	455 ± 5 ^l^	77 ± 4 ^f^	4 ± 0 ^abc^	53 ± 3 ^j^	142 ± 10 ^c^	47 ± 2 ^ab^	130 ± 5 ^de^	1187 ± 29 ^ef^
***Pulp***											
**Summer Prince**	48 ± 1 ^K^	121 ± 1 ^H^	4 ± 0 ^A^	427 ± 6 ^K^	n.d.	n.d.	n.d.	6 ± 0 ^K^	6 ± 0 ^F^	n.d.	611 ± 6 ^H^
**Ruby-S**	53 ± 0 ^L^	6 ± 0 ^A^	n.d.	652 ± 6 ^P^	n.d.	n.d.	3 ± 0 ^A^	8 ± 0 ^M^	n.d.	n.d.	722 ± 7 ^J^
**Summer King**	14 ± 0 ^A^	4 ± 0 ^A^	n.d.	243 ± 5 ^F^	n.d.	n.d.	n.d.	6 ± 0 ^GHIJ^	5 ± 0 ^C^	n.d.	272 ± 5 ^BC^
**Tinkerbell**	89 ± 3 ^P^	4 ± 0 ^A^	n.d.	1623 ± 3 ^U^	10 ± 0 ^A^	n.d.	4 ± 0 ^B^	6 ± 0 ^J^	5 ± 0 ^D^	n.d.	1741 ± 6 ^O^
**Arisoo**	28 ± 1 ^DE^	5 ± 0 ^A^	n.d.	215 ± 3 ^D^	n.d.	n.d.	n.d.	5 ± 0 ^DEF^	n.d.	n.d.	253 ± 4 ^AB^
**Decobell**	53 ± 1 ^L^	298 ± 2 ^L^	9 ± 0 ^L^	268 ± 3 ^G^	n.d.	n.d.	4 ± 1 ^B^	7 ± 0 ^L^	n.d.	n.d.	639 ± 6 ^I^
**Green Ball**	30 ± 3 ^FG^	80 ± 10 ^D^	4 ± 0 ^B^	142 ± 17 ^B^	n.d.	n.d.	n.d.	5 ± 0 ^CDE^	5 ± 0 ^D^	n.d.	267 ± 30 ^BC^
**Honggeum**	44 ± 1 ^J^	100 ± 2 ^F^	4 ± 0 ^D^	562 ± 8 ^N^	n.d.	n.d.	n.d.	n.d.	6 ± 0 ^E^	n.d.	717 ± 10 ^J^
**Hwangok**	125 ± 2 ^Q^	100 ± 1 ^F^	7 ± 0 ^K^	231 ± 2 ^EF^	n.d.	n.d.	n.d.	6 ± 0 ^IJ^	n.d.	n.d.	469 ± 5 ^E^
**Picnic**	84 ± 2 ^O^	83 ± 2 ^D^	6 ± 0 ^H^	1183 ± 23 ^T^	n.d.	n.d.	n.d.	5 ± 0 ^BCD^	5 ± 0 ^B^	n.d.	1367 ± 27 ^M^
Gala	29 ± 1 ^EF^	98 ± 2 ^EF^	6 ± 0 ^I^	523 ± 11 ^M^	n.d.	n.d.	n.d.	n.d.	n.d.	n.d.	657 ± 14 ^I^
Sansa	71 ± 0 ^N^	113 ± 1 ^G^	5 ± 0 ^E^	775 ± 2 ^R^	n.d.	n.d.	n.d.	6 ± 0 ^EFG^	5 ± 0 ^A^	n.d.	974 ± 3 ^L^
Tsugaru	23 ± 0 ^C^	93 ± 1 ^E^	n.d.	125 ± 1 ^A^	n.d.	n.d.	n.d.	5 ± 0 ^ABC^	n.d.	n.d.	246 ± 1 ^A^
Hongro	70 ± 2 ^N^	130 ± 2 ^I^	4 ± 0 ^C^	755 ± 4 ^Q^	n.d.	n.d.	n.d.	6 ± 0 ^HIJ^	6 ± 0 ^E^	n.d.	971 ± 6 ^L^
Sekaiichi	70 ± 3 ^N^	140 ± 6 ^J^	5 ± 0 ^D^	225 ± 9 ^DE^	n.d.	n.d.	n.d.	6 ± 0 ^DEFG^	8 ± 0 ^H^	n.d.	452 ± 18 ^E^
Alps Otome	29 ± 1 ^DEF^	221 ± 10 ^K^	4 ± 0 ^AB^	1164 ± 13 ^S^	n.d.	n.d.	n.d.	6 ± 0 ^IJ^	n.d.	n.d.	1424 ± 24 ^N^
Golden Delicious	19 ± 0 ^B^	85 ± 1 ^D^	4 ± 0 ^AB^	349 ± 1 ^H^	n.d.	n.d.	n.d.	6 ± 0 ^FGH^	6 ± 0 ^G^	n.d.	468 ± 2 ^E^
Jonagold	40 ± 0 ^I^	98 ± 1 ^EF^	5 ± 0 ^F^	394 ± 3 ^J^	n.d.	n.d.	n.d.	6 ± 0 ^HIJ^	8 ± 0 ^I^	n.d.	551 ± 4 ^G^
Jonathan	38 ± 1 ^H^	141 ± 4 ^J^	7 ± 0 ^J^	461 ± 10 ^L^	n.d.	n.d.	n.d.	7 ± 0 ^L^	n.d.	n.d.	653 ± 14 ^I^
Kamhong	57 ± 1 ^M^	48 ± 1 ^B^	4 ± 0 ^D^	167 ± 2 ^C^	n.d.	n.d.	n.d.	6 ± 0 ^FGHI^	n.d.	n.d.	282 ± 4 ^C^
Yoko	32 ± 0 ^G^	132 ± 1 ^I^	5 ± 0 ^G^	163 ± 1 ^C^	n.d.	n.d.	n.d.	5 ± 0 ^A^	n.d.	n.d.	338 ± 3 ^D^
Fuji	19 ± 0 ^B^	99 ± 2 ^EF^	n.d.	375 ± 5 ^I^	n.d.	n.d.	n.d.	5 ± 0 ^AB^	n.d.	n.d.	498 ± 7 ^F^
Indo	14 ± 0 ^A^	68 ± 1 ^C^	n.d.	238 ± 4 ^EF^	n.d.	n.d.	n.d.	n.d.	n.d.	n.d.	320 ± 5 ^D^
Ralls Janet	27 ± 1 ^D^	108 ± 2 ^G^	4 ± 0 ^AB^	620 ± 12 ^O^	n.d.	n.d.	n.d.	5 ± 0 ^ABC^	n.d.	n.d.	764 ± 15 ^K^

Bold letters indicate new early- to mid-season apple cultivars. Mean values followed by different lower case and capital letters indicate significant cultivar differences for peel or pulp, respectively, at *p* < 0.05. Cat, Epi, Rut, Q3gal, Q3glc, Q3ara, Q3rha, Phl, CA, C3gal, and C3ara indicate catechin, epicatechin, rutin, quercetin-3-*O*-galactoside, quercetin-3-*O*-glucoside, quercetin-3-*O*-arabinoside, quercetin-3-*O*-rhamnoside, phloridzin, chlorogenic acid, cyanidin-3-*O*-galactoside, and cyanidin-3-*O*-arabinoside, respectively.

## Supplementary Materials

The following are available online at https://www.mdpi.com/2076-3921/9/5/443/s1, Figure S1: Extracted ion chromatogram (EIC) of the identified phenolic compounds by UHPLC-(ESI)-qTOF in the peel (a) and pulp (b) of Decobell apples. Peak numbers are annotated in Table S1, Table S1: Phenolic compounds identified by UHPLC-(ESI)-qTOF in the apple peel and pulp, Table S2: F-values and *p*-values in the analysis of variance for metabolites among the peel and pulp of apple cultivars.
