# Supplementary material for: Metabolite Profiling and Antioxidant Activity of 10 New Early- to Mid-Season Apple Cultivars and 14 Traditional Cultivars
Source: Antioxidants (Basel). 2020 May 20;9(5):443. doi: 10.3390/antiox9050443 (PMC7278836; doi:10.3390/antiox9050443)
Supplement: Supplementary file 1 [file antioxidants-09-00443-s001.pdf]

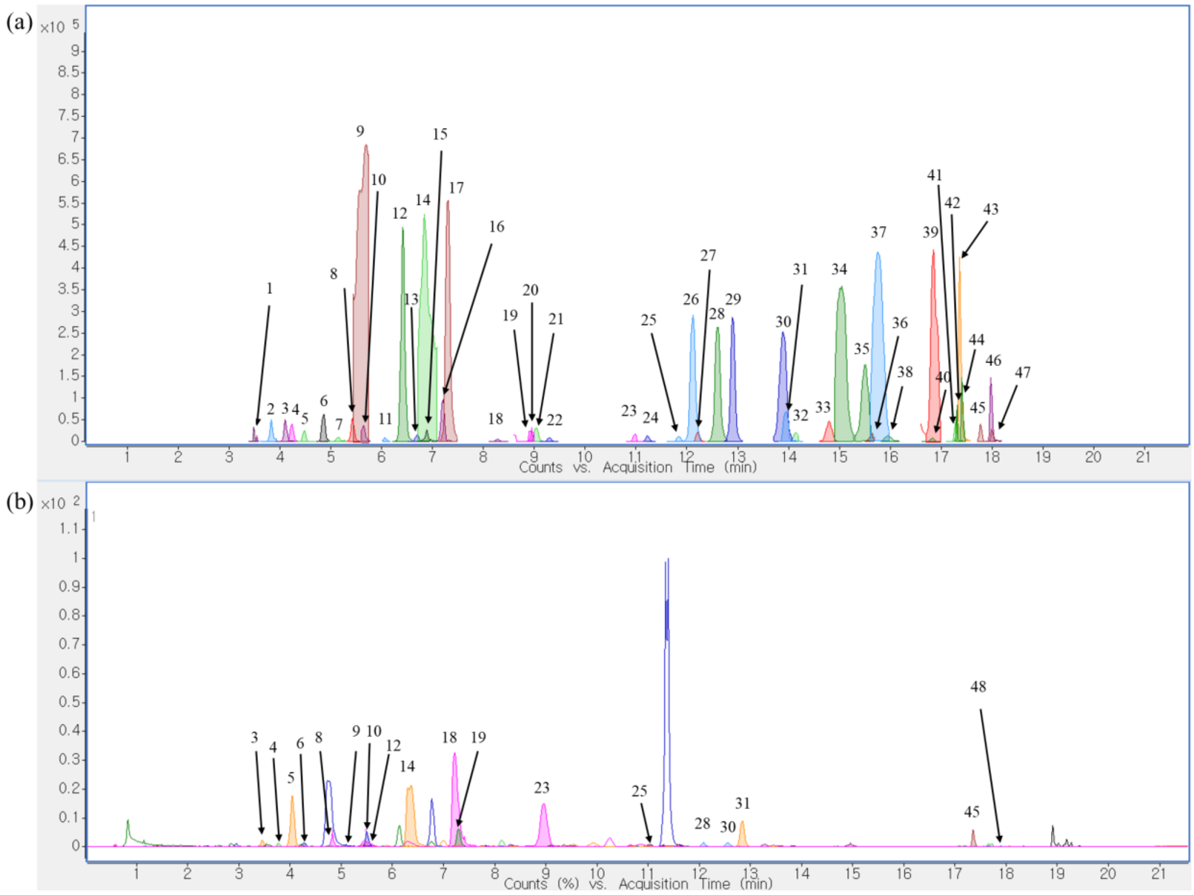

Figure S1. Extracted ion chromatogram (EIC) of the identified phenolic compounds by UHPLC-(ESI)-qTOF in the peel (a) and pulp (b) of Decobell apples. Peak numbers are annotated in Table S1.

Table S1. Phenolic compounds identified by UHPLC-(ESI)-qTOF in the apple peel and pulp

Code (peak no.)	Rt (min)	Compound assigned	Molecular formula	Predicted MS1 m/z	Fragment m/z	Error (ppm)	Subclass	Found in peel or pulp
Phenolic acid								
PA1 (1)	3.5	hydroxy benzoic acid	$C_7H_6O_3$	137.0244	119 $[M - H_2O - H]^-$<br>108 $[M - CO (-28) - H]^-$	-5.1	hydroxybenzoic acid	peel, pulp
PA2 (15)	6.9	dihydroxy benzoic acid	$C_7H_6O_4$	153.0193	137 $[M - CO (-28) - H]^-$<br>109 $[M - CO_2 (-44) - H]^-$	-0.8	hydroxybenzoic acid	peel
PA3 (4)	4.2	chlorogenic acid*	$C_{16}H_{18}O_9$	353.0878	191 $[M - caf (-162) - H]^-$<br>179 $[M - qa (-174) - H]^-$	-2.0	hydroxycinnamic acid	peel, pulp
PA4 (8)	5.4	caffeoylquinic acid	$C_{16}H_{18}O_9$	353.0878	191 $[M - caf (-162) - H]^-$<br>179 $[M - qa (-174) - H]^-$	0.3	hydroxycinnamic acid	peel, pulp
PA5 (13)	6.7	coumaroylquinic acid	$C_{16}H_{18}O_8$	337.0929	191 $[M - cu (-146) - H]^-$<br>173 $[M - cu (-146) - H_2O - H]^-$	0.1	hydroxycinnamic acid	peel
PA6 (16)	7.2	coumaroylquinic acid	$C_{16}H_{18}O_8$	337.0929	191 $[M - cu (-146) - H]^-$	0.5	hydroxycinnamic acid	peel, pulp
PA7 (20)	8.9	coumaroylquinic acid	$C_{16}H_{18}O_8$	337.0929	191 $[M - cu (-146) - H]^-$	-2.9	hydroxycinnamic acid	peel
Flavan-3-ols								
P1 (2)	3.8	catechin-*O*-hexoside	$C_{21}H_{24}O_{11}$	451.1246	289 $[M - h (-162) - H]^-$	-3.3	flavan-3-ol	peel, pulp
P2 (3)	4.1	procyanidin dimer B type	$C_{30}H_{26}O_{12}$	577.1351	289 $[M - cat (-288) - H]^-$	-2.3	proanthocyanidins	peel, pulp
P3 (5)	4.5	catechin-*O*-hexoside	$C_{21}H_{24}O_{11}$	451.1246	289 $[M - h (-162) - H]^-$<br>245 $[M - h (-162) - C_2H_4O - H]^-$	3.6	flavan-3-ol	peel
P4 (6)	4.9	catechin*	$C_{15}H_{14}O_6$	289.0718	203 $[M - C_4H_6O_2 - H]^-$<br>151 $[M - C_7H_6O_3 - H]^-$<br>109 $[M - C_9H_8O_4 - H]^-$	-1.2	flavan-3-ol	peel, pulp
P5 (7)	5.1	(epi)gallocatechin	$C_{15}H_{14}O_7$	305.0667		1.8	flavan-3-ol	peel, pulp
P6 (10)	5.7	procyanidin trimer B type	$C_{45}H_{38}O_{18}$	865.1985		1.9	proanthocyanidins	peel, pulp
P7 (11)	6.1	(epi)gallocatechin	$C_{15}H_{14}O_7$	305.0667	125 $[M - C_9H_8O_4 - H]^-$	0.1	flavan-3-ol	peel
P8 (12)	6.4	procyanidin dimer B type	$C_{30}H_{26}O_{12}$	577.1351	289 $[M - cat (-288) - H]^-$	0.0	proanthocyanidins	peel, pulp
P9 (17)	7.3	epicatechin*	$C_{15}H_{14}O_6$	289.0718	245 $[M - CO_2 (-44) - H]^-$	1.5	flavan-3-ol	peel, pulp
P10 (21)	9.0	procyanidin trimer B type	$C_{45}H_{38}O_{18}$	865.1985	577 $[M - cat (-288) - H]^-$<br>289 $[M - cat (-288) - cat (-288) - H]^-$	-3.7	proanthocyanidins	peel, pulp

P11 (29)	12.9	procyanidin dimer B type	$C_{30}H_{26}O_{12}$	577.1351	289 [M – cat (–288) – H]⁻	3.2	proanthocyanidins	peel, pulp
P12 (33)	14.8	catechin-*O*-hexoside	$C_{21}H_{24}O_{11}$	451.1246	289 [M – h (–162) – H]⁻	-1.5	flavan-3-ol	peel
Flavonols								
F1 (18)	8.3	myricetin-*O*-hexoside	$C_{21}H_{20}O_{13}$	479.0831	317 [M – h (–162) – H]⁻	-0.7	flavonol	peel
F2 (22)	9.3	quercetin-*O*-dihexoside	$C_{27}H_{30}O_{17}$	625.141	463 [M – h (–162) – H]⁻<br>301 [M – h (–162) –h (–162) – H]⁻	0.2	flavonol	peel
F3 (24)	11.2	quercetin-*O*-pentose hexoside	$C_{26}H_{28}O_{16}$	595.1305	300 [M – h (–162) –p (–132) – H]⁻	-0.8	flavonol	peel
F4 (26)	12.1	quercetin-3-*O*-galactoside*	$C_{21}H_{20}O_{12}$	463.0882	301 [M – h (–162) – H]⁻	0.9	flavonol	peel, pulp
F5 (27)	12.2	rutin*	$C_{27}H_{30}O_{16}$	609.1461	447 [M – h (–162) – H]⁻<br>301 [M – h (–162) –dh (–146) – H]⁻	-0.1	flavonol	peel
F6 (28)	12.6	quercetin-3-*O*-glucoside*	$C_{21}H_{20}O_{12}$	463.0882	300 [M – h (–162) – H]⁻	-0.8	flavonol	peel, pulp
F7 (30)	13.9	quercetin-3-*O*-xyloside*	$C_{20}H_{18}O_{11}$	433.0776	300 [M – p (–132) – H]⁻	-1.8	flavonol	peel
F8 (32)	14.1	quercetin-*O*-rhamnoside	$C_{21}H_{20}O_{11}$	447.0933	300 [M – dh (–146) – H]⁻	-3.2	flavonol	peel
F9 (34)	15.1	quercetin-*O*-pentoside	$C_{20}H_{18}O_{11}$	433.0776	301 [M – p (–132) – H]⁻	-0.7	flavonol	peel
F10 (35)	15.5	quercetin-3-*O*-arabinoside*	$C_{20}H_{18}O_{11}$	433.0776	301 [M – p (–132) – H]⁻	-0.4	flavonol	peel
F11 (36)	15.6	isorhamnetin-*O*-hexoside	$C_{22}H_{22}O_{12}$	477.1038	315 [M – h (–162) – H]⁻	-2.1	flavonol	peel
F12 (37)	15.8	quercetin-3-*O*-rhamnoside*	$C_{21}H_{20}O_{11}$	447.0933	301 [M – dh (–146) – H]⁻	-2.4	flavonol	peel
F13 (38)	16.0	isorhamnetin-*O*-hexoside	$C_{22}H_{22}O_{12}$	477.1038		-2.8	flavonol	peel
F14 (40)	16.8	isorhamnetin-3-*O*-glucoside*	$C_{22}H_{22}O_{12}$	477.1038	314 [M – h (–162) – H]⁻	-0.9	flavonol	peel
F15 (42)	17.3	myricetin-*O*-hexoside	$C_{21}H_{20}O_{13}$	479.0831	317 [M – h (–162) – H]⁻	0.3	flavonol	peel
F16 (45)	17.8	quercetin deoxyhexose hexoside	$C_{30}H_{26}O_{14}$	609.125	463 [M – dh (–146) – H]⁻<br>301 [M – dh (–146) – h (–162) – H]⁻	0.1	flavonol	peel
F17 (46)	18.0	quercetin*	$C_{15}H_{10}O_7$	301.0354		-2.5	flavonol	peel, pulp
F18 (47)	18.0	kaempferol*	$C_{15}H_{10}O_6$	285.0405		1.7	flavonol	peel
Flavanones								
V1 (23)	11.0	naringenin-*O*-hexoside	$C_{21}H_{22}O_{10}$	433.1140	271 [M – h (–162) – H]⁻	1.0	flavanone	peel, pulp
V2 (25)	11.8	eriodictyol-7-*O*-glucoside*	$C_{21}H_{22}O_{11}$	449.1089	287 [M – h (–162) – H]⁻	0.9	flavanone	peel
Anthocyanins								

A1 (9)	5.6	cyanidin-3-*O*-galactoside*	$C_{21}H_{20}O_{11}$	449.1078+	287 [M-h (–162) + H]$^{+}$	-3.4	anthocyanin	peel, pulp
A2 (14)	6.9	cyanidin-3-*O*-arabinoside*	$C_{20}H_{18}O_{10}$	419.0973+	287 [M–h (–132) + H]$^{+}$	-0.6	anthocyanin	peel
A3 (19)	8.8	cyanidin-*O*-pentoside	$C_{20}H_{18}O_{10}$	419.0973+	287 [M – p (–132) + H]$^{+}$	1.1	anthocyanin	peel
A4 (31)	14.0	cyanidin-*O*-hexoside	$C_{21}H_{20}O_{11}$	449.1078+	287 [M – h (–162) + H]$^{+}$	0.3	anthocyanin	peel
A5 (41)	17.3	cyanidin-*O*-pentoside	$C_{20}H_{18}O_{10}$	419.0973+	287 [M – h (–132) + H]$^{+}$	-1.7	anthocyanin	peel
A6 (44)	17.4	cyanidin-*O*-rhamnoside*	$C_{21}H_{20}O_{10}$	433.1129+	287 [M – dh (–146) + H]$^{+}$	-2.0	anthocyanin	peel
Dihydrochalcone								
PH1 (39)	16.8	phloretin-*O*-pentose hexoside	$C_{26}H_{32}O_{14}$	567.1719	273 [M – h (–162) –p (–132) – H]$^{-}$	0.9	dihydrochalcone	peel
PH2 (43)	17.4	phloridzin*	$C_{21}H_{24}O_{10}$	435.1297	273 [M – h (–162) – H]$^{-}$	-0.5	dihydrochalcone	peel, pulp

* identified with authentic standards.

+ indicates that the values were measured in ESI-positive mode and other m/z values were measured in ESI-negative mode.

qa-quinic, caf-caffeoyl, cu-coumaroyl, cat-catechin, h-hexose, dh-deoxyhexose, p-pentose.

Table S2. F-values and *p*-values in the analysis of variance for metabolites among the peel and pulp of apple cultivars

	**Frc**	**Glc**	**Suc**	**Sor**	**Total free sugar**	**Mal**	**Cit**	**Shi**	**Total organic acid**	**Sugar/acid**	**DPPH**	**ABTS**	**QTof EIC area**	**Cat**	**Epi**	**Phl**	**CA**	**C3gal**	**C3 ara**	**Rut**	**Q3gal**	**Q3glc**	**Q3 rha**	**Total phenolic**
F value																								
Peel	-	-	-	-	-	-	-	-	-	-	1834	240	148	321	61	199	5641	294	331	576	168	267	241	91
Pulp	1233	42	1283	447	231	4585	109541	39	5434	1517	121	423	324	1332	1166	2523	6164	14048	n.d.	103	1306	5404	n.d.	3175
p-value																								
Peel	-	-	-	-	-	-	-	-	-	-	**	**	**	**	**	**	**	**	**	**	**	**	**	**
Pulp	**	**	**	**	**	**	**	**	**	**	**	**	**	**	**	**	**	**	n.d.	**	**	**	n.d.	**

Frc, Glc, Suc, Sor, Mal, Cit, Shi, Cat, Epi, Phl, CA, C3gal, C3ara, Rut, Q3gal, Q3glc, and Q3rha indicate fructose, glucose, sucrose, sorbitol, malic acid, citric acid, shikimic acid, catechin, epicatechin, phloridzin, chlorogenic acid, cyanidin-3-*O*-galactoside, cyanidin-3-*O*-arabinoside, rutin, quercetin-3-*O*-galactoside, quercetin-3-*O*-glucoside, and quercetin-3-*O*-rhamnoside, respectively. –, n.d., and ** indicate not determined, not detected, and significant differences at $p < 0.01$, respectively.
